# Dietary protein, lipid and insect meal on growth, plasma biochemistry and hepatic immune expression of lake whitefish (*Coregonus clupeaformis*)

**DOI:** 10.1016/j.fsirep.2023.100111

**Published:** 2023-07-07

**Authors:** Yubing Chen, Rebecca Lawson, Umesh Shandilya, Marcia A. Chiasson, Niel A. Karrow, David Huyben

**Affiliations:** a Department of Animal Biosciences, University of Guelph, Guelph, ON, Canada; b Ontario Aquaculture Research Centre, Office of Research, University of Guelph, Elora, ON, Canada

**Keywords:** Fish feed, Hermetia illucens, Nutrition, qPCR, Salmonids

## Abstract

Studies are lacking that investigate the dietary nutrient requirements of lake whitefish (*Coregonus clupeaformis*), a newly farmed fish species in Ontario, Canada. Dietary levels of protein and lipid must be optimized to ensure high growth performance for the commercial success of this species. Additionally, the inclusion of insect meal in the diet may improve growth and immune response. The objective of this study was to evaluate the effects of dietary protein:lipid ratios and insect meal as a feed additive on the growth performance and hepatic immune function of juvenile lake whitefish (301 ± 10 g). A 16-week (112 day) trial was performed with five diets including a commercial control diet (BCC), and four experimental diets with high or low levels of protein (54 and 48%, respectively) and lipid (18 and 12%, respectively). The high protein dietary groups contained 5% of full-fat black soldier fly larvae (*Hermetia illucens*). Fish weights, viscera, liver, and blood were collected for further analysis. Specific growth rate, thermal growth coefficient and weight gain were significantly higher in fish fed with the BCC and high protein high lipid (HPHL) diets. However, viscerosomatic index was found to be significantly higher in fish fed the BCC diet, thus HPHL is more optimal for non-visceral weight gain. Higher levels of plasma phosphorus, aspartate aminotransferase and potassium indicated poor growth and stress in fish fed low lipid diets. Relative expression of *HSP70*, involved in cellular repair, was significantly downregulated in fish fed high lipid diets, and no effects were found on the expression of innate immune and oxidative stress genes. Also, *IL8 (CXCL8)* and *catalase* were upregulated (non-significant) in fish fed the HPHL diet with the largest weight gain. No effects of insects were found on growth, plasma biochemistry or gene expression, which suggests 5% dietary inclusion was too low. Overall, we recommend a HPHL diet for the cultivation of lake whitefish based on improved growth performance, low viscera weight, improved plasma biochemistry and downregulation of cellular repair genes.

## 1 Introduction

Lake whitefish (*Coregonus clupeaformis*) is a newly farmed fish species in Ontario, Canada. Interest in the aquacultural production of this species has increased in recent years due to the limited wild fisheries production. Additionally, some populations of lake whitefish are considered threatened [1]. Sustainable commercial production of lake whitefish would not only maintain a supply for human consumption, but would also reduce the fishing pressure on wild stocks. Lake whitefish are a cold-water species in the salmonid family (Salmonidae) with a total length and weight up to 65 cm and 1.8 kg, respectively, and they typically feed on benthic macroinvertebrates on the bottom of freshwater lakes [1,2]. Lake whitefish is an important food source for the Indigenous people of North America as they have fished them from the Great Lakes for many centuries [2]. Unlike other farmed salmonid fishes, limited nutritional studies have been conducted on lake whitefish. To date, a commercial diet for lake whitefish does not exist in Canada and they are commonly fed diets optimized for other salmonids, e.g. rainbow trout. Therefore, it is crucial to investigate the nutritional requirement of lake whitefish to better facilitate optimal growth and production in aquaculture systems.

Optimizing protein:lipid ratios in fish diets are also considered an important factor to maintain immune system function, increase disease resistance, and promote growth performance. Dietary lipid is an important nutritional factor that affects the innate immune function since immune cells are comprised of lipids, e.g. the lipid bilayer in the cellular membrane [3]. Omega-3 polyunsaturated fatty acids, such as arachidonic acid, are also converted to eicosanoids that help signal cells during the immune response [4,5]. Inadequate and imbalanced levels of total dietary lipid and fatty acids may affect fish immunity because of the impaired immune cell membrane, which may lead to a malfunctioning innate immune system [3]. For instance, a previous study found that the plasma lysozyme activity of juvenile grass carp (*Ctenopharyngodon idella*) was positively correlated with the increasing dietary lipid level from 0% to 10%, and the survival rate of the juvenile grass carp challenged with *Aeromonas hydrophila* was the highest when fed with 7.5% of dietary lipid [6]. In addition, dietary protein provides essential amino acids and nitrogen required for animal growth, metabolism, and many bodily functions [7]. Adequate levels of dietary protein and amino acids in fish diets are important, since amino acids are required for the synthesis of bioactive proteins that participate in the defense mechanisms, disease resistance, and key immune regulatory pathways that are important for fish health [7].

Insects and benthic macroinvertebrates are considered part of the natural diet of many carnivorous and omnivorous fish species. Feeding locally produced insects to fish has resulted in lower ecological footprints, thereby reducing the pressure on fishmeal and fish oil while increasing the sustainability of the aquaculture industry [8], [9], [10]. More importantly, chitin in the exoskeleton of insects serves as an important immunostimulant since it has been found to boost innate immunity and improve stress response in fish [11]. Several studies have demonstrated that feeding insect meal, such as black soldier fly larvae (BSFL), to rainbow trout resulted in higher fish growth performance, feed intake, and gut bacterial diversity [12], [13], [14], [15]. A study by Kumar et al. [16] found an upregulation of interferon regulatory factor-1 (IRF-1) in the kidney of rainbow trout (*Oncorhynchus mykiss*) when fed 8% inclusion of BSFL compared to diets with fish meal and soybean meal. Additionally, the survival rate of European seabass (*Dicentrarchus labrax*) improved when fed with BSFL meal and challenged with *Vibrio alginolyticus*, which suggests BSFL meal increased disease resistance and plays a role in immune function [17,18]. However, few studies have investigated the effects of feeding BSFL on the innate immune response in fish, especially lake whitefish.

We hypothesized that the high lipid, high protein diets and the addition of BSFL as a feed additive increases growth performance and upregulates the innate immune response of lake whitefish due to increased levels of fatty acids available to produce immune cells and their natural diet of insects in the wild. The main objective of this study was to investigate the effects of dietary protein:lipid ratios on the growth, feed conversion, body indices, plasma biochemistry, and the hepatic gene expression of innate immune, cellular repair and oxidative stress responses of lake whitefish after a 16-week feeding trial. The second objective was to examine the effects of insect meal (BSFL) as a feed additive.

## 2 Materials and methods

### 2.1 Fish, facilities and diets

Lake whitefish fry were hatched from fertilized eggs collected from wild adults in Georgian Bay and were transported to the Ontario Aquaculture Research Centre (Alma, ON, Canada). A total of 450 mixed sexed, lake whitefish (301 ± 10 g) were randomly distributed across 15 one-meter diameter fiberglass tanks where each tank contained 30 fish. Fish were acclimated for 14 days in the experimental tanks, during which time they were fed a high-quality commercial rainbow trout diet (Bluewater Feed 48-18, Sharpe Farm Supplies, Guelph, ON, Canada) twice daily. During the experiment, fish were fed to satiety over a two-hour period (9:00-11:00) in the morning and a two-hour period (14:00-16:00) in the afternoon for two days per week and the remaining days the fish were fed using automatic belt feeders with the average amount from the previous two hand feeding days. All procedures involving the handling and treatment of fish used in this study were approved by the Animal Care Committee at the University of Guelph under Animal Utilization Protocol #4586.

Water quality was measured for temperature, dissolved oxygen, and total suspended solids every two weeks. The water temperature was constant at 8.5 °C, dissolved oxygen level was 10.4 mg/L and the mean total suspended solids of inflow water was 0.1 mg/L. The experimental photoperiod was 12:12 light to darkness utilizing LED lights with a 60-min ramp time to simulate dawn and dusk. Water flow was maintained at 11 L/min.

Triplicate tanks (n=3) were set up as a randomized block design, and each tank was fed one of five diets for 112 days that contained low and high levels of protein (48 and 54%) or lipid (12 and 18%), with an additional Bluewater commercial control (BCC) in a 2 × 2 + 1 design. The high protein diets contained 5% full-fat black soldier fly larvae (Oreka Solutions, Cambridge, ON, Canada). The diets were formulated to have the same level of energy (isoenergetic) with a gross energy between 21 and 22 MJ/kg. All feed ingredients were ground to <1 mm, mixed for 20 min with the dry ingredients followed by another 20 min with the wet ingredients (Table 1). The mash was pre-conditioned to 65 °C, steam pelleted (California Pellet Mill Co., IN, USA) and dried overnight (16 h) at 40°C. Pellets were 3 mm in diameter, sieved to remove fines and stored at 4°C until the trial started.

**Table 1:** Diet formulation (g/kg).

	Bluewater Commercial Control	Low protein + High Lipid*	Low Protein + Low Lipid	High Protein + High Lipid	High Protein + Low Lipid
	BCC	LPHL*	LPLL	HPHL	HPLL
Bluewater feed 48-18	950				
Fish meal, herring		200	200	200	200
Poultry by-product meal		250	250	250	250
Corn gluten		100	100	100	100
Wheat flour		100	100	100	100
Wheat gluten	50	50	50	50	50
Blood meal		50	50	50	50
Soy protein HP300		80	80	105	105
BSFL full-fat meal				50	50
Corn starch		60	116		56
Canola oil		50	22	35	7
Fish oil		50	22	50	22
Vitamin & mineral premix		5	5	5	5
Calcium carbonate		4	4	4	4
Salt		1	1	1	1

BSFL: black soldier fly larvae.

⁎ LPHL diet was used as a control diet in this experiment since the BCC diet was a commercial control.

### 2.2 Sample collection

At start and end of the trial, all fish were sedated with tricaine methanesulfonate (MS222; Syndel Canada) and individually weighed (g) and measured for fork length (cm). At the start, nine fish from the stock population were dissected to measure body indices. Viscera, liver, and fat separated from viscera in each fish were removed and weighed, respectively, to calculate body indices. For the duration of the trial, the biomass of each tank was assessed via bulk weighing every 28 days. Feed intake was recorded daily to calculate feed conversion ratio (FCR). At the end of the trial, six fish per tank were euthanized with an overdose of MS222 followed by cervical dislocation. Fish were first measured for individual body weight and fork length. Two millilitres of blood were collected from the caudal vein using heparinized syringes, centrifuged at 2000 *g* for 2 min and the plasma was stored at -80 °C. Fish were dissected and the viscera, liver, and fat were weighed for body indices (*n* = 6 per diet). From three fish per tank, 1 cm of liver apex was collected into a sterile cryovial tube, placed on dry ice and stored at -80 °C (*n* = 9 per diet).

### 2.3 Growth performance analyses

Fish body weight was recorded at the start and end of the trial to calculate weight gain (WG = (Final body weight – Initial body weight) / Initial body weight *100), specific growth rate (SGR = [ln Final body weight (g) – ln Initial body weight (g)] / days *100), and thermal growth coefficient (TGC = (Final body weight^1/3^ – Initial body weight^1/3^) / (Temperature*day) *1000). Feed intakes (FI) were used to calculate feed conversion ratio (FCR = Feed intake (g) / Fish weight gain (g). Viscera weight, liver weight and the visceral fat weight were recorded to calculate the Viscerosomatic index (VSI = Viscera weight (g) / Body weight (g) *100), Hepatosomatic index (HSI = Liver weight (g) / Body weight (g) *100) and Liposomatic index (LSI = Visceral fat weight (g) / Body weight (g) *100).

### 2.4 Plasma biochemistry analyses

Plasma biochemistry analyses were performed by the Animal Health Laboratory at the University of Guelph (Guelph, ON, Canada) using Cobas c501 module (Roche Diagnostic, Basel, Switzerland). Two hundred microliters of lithium-heparin plasma were pooled from 3 fish per tank and there were three tanks per diet (*n* = 9 per diet). The mid volume analyzer was comprised of a photometric unit for a broad range of clinical chemistry assays of total protein, albumin, globulin, glucose, cholesterol, aspartate aminotransferase, creatine kinase, amylase, lactate dehydrogenase, bile acid, glutamate dehydrogenase, carbon dioxide, lipase, uric acid and urea, and an ISE unit for ion-selective electrode determinations of sodium, potassium, chloride, calcium, and phosphorus.

### 2.5 RNA extraction and cDNA synthesis

30 mg of liver was placed into tubes that contained 0.1 g 1.0 mm silica beads (Sigma-Aldrich, Darmstadt, Germany) and 600 µL RLT buffer (Qiagen NV, Hilden, Germany). Samples were homogenized using Qiagen TissueLyzer II (Qiagen NV) for 2 cycles of 2 min at a speed of 30 Hz and then centrifuged for 3 min at 16,000 g at 4 °C using Sorvall Legend Micro 21R centrifuge (Thermo Fisher Scientific, Waltham, MA, USA). The supernatant was transferred into spin columns for mRNA extraction using the RNeasy Mini Kit (Qiagen NV) according to the manufacturer's protocol. The quantity and purity of RNA were assessed using BioTek Cytation 5, Cell Imaging Multi-Mode Reader (Agilent, Santa Clara, United States). RNA samples had an average 260/280 nm absorbance ratio of 2.14 ± 0.017 that the RNA samples with an absorbance ratio lower than 2.00 was performed with RNA extraction again until it reaches our desired threshold. The RNA samples were then diluted with nuclease-free water to obtain 1000 ng of RNA. cDNA synthesis was completed using high-capacity cDNA reverse transcription kit (Applied Biosystems, Waltham, United State) in 20 µL reaction volumes, comprising 10 µL RNA sample, 2 µL RT buffer, 0.8 µL dNTP, 2 µL random primer, 1 µL reverse transcriptase and 4.2 µL nuclease-free water according to the manufacturer's instructions. PCR conditions for cDNA synthesis included one cycle of 25 °C for 10 min, 37 °C for 120 min and 85°C for 5 min using MiniAmp Thermo Cycler (Applied Biosystems). The cDNA samples were then diluted 1:5 to obtain 100 µL volumes.

### 2.6 Gene expression by RT-qPCR

The forward and reverse primers for each gene used are listed in Table 2. Primer sequences were selected using BLAST (NCBI) by comparing between the predicted genes of lake whitefish with the sequence data from Atlantic salmon and rainbow trout. The accession numbers have been provided in Table 2, as they may not represent the putative target gene due to evolutionary chromosome duplication events (tetraploidization). Primers were checked using gel electrophoresis through each DNA band by comparing to their theoretical amplicon sizes. Primers were diluted with nuclease-free water to a concentration of 10 µM. Duplicates were prepared for each sample and pipetted into each well in a 10 µL reaction comprising of 2 µL cDNA sample, 2 µL nuclease-free water, 0.5 µL forward primer, 0.5 µL reverse primer and 5 µL SsoAdvanced Universal SYBR Green Supermix (Bio-Rad Laboratories Inc, Hercules, United States). The plates were then centrifuged for 4 min at 1500 rpm. RT-qPCR conditions included holding at 95 °C for 10 min, 40 cycles of 95 °C for 15 s and 60 °C for 1 min followed by a melt curve at 95°C for 15 s, 60°C for 1 min, 95°C for 15 s using the StepOnePlus Real-Time PCR system (Applied biosystems). Only β-actin and EF-1α were used as references genes since β-actin and EF-1α were more stable than 18S rRNA according to the RefFinder software [19] and were not affected by treatments. The targeted genes were normalized using the geometric mean of the C_T_ values from the control LPHL diet: ΔC_T_ = Calibrator C_T_ - Sample C_T_. The relative quantity (RQ) was calculated as RQ = (Primer Efficiency) ^ΔC_T._ The relative expression was then calculated as RQ_GOI_/ GEOMEAN[RQ_Ref_] according to the method provided by Vandesompele et al. [20] (Average primer efficiency=2.087).

**Table 2:** Primer pair information of target and reference genes related to pro- and anti-inflammatory cytokines, heat shock proteins, and oxidative stress proteins (annealing temp 60 °C).

Function	Gene	Amplicon size (bp)	Primer	Sequence (5’-3’)	Accession number
Housekeeping genes	*β-actin*	155	Forward	GGAAGATGAAATCGCCGCAC	AB196465
			Reverse	AGCTGTCTTTCTGGCCCATC	
	*18S rRNA*	106	Forward	CCCAAATCAAGTCCAATTCACA	XM_021588520.1
			Reverse	CTGTCTTCTCCTCCCCTCCA	
	*EF-1α*	62	Forward	GGCAAGAAACTTGAGGATGC	AF498320
			Reverse	ACAGTCTGCCTCATGTCACG	
Pro-inflammatory Cytokines	*TNFα*	208	Forward	CAAFAFTTTFAACCTCATTCAG	AJ401377
			Reverse	GCTGCTGCCGCACATAAAG	
	*IL1β*	181	Forward	ACCGAGTTCAAGGACAAGGA	AJ223954
			Reverse	CATTCATCAGGACCCAGCAC	
Anti-inflammatory Cytokine	*TGFβ*	275	Forward	AGATAAATCGGAGAGTTGCTGTG	AJ007836
			Reverse	CCTGCTCCACCTTGTGTTGT	
Chemokine	*IL8* (*CXCL8*)	162	Forward	CACAGACAGAGAAGGAAGGAAAG	AJ279069
			Reverse	TGCTCATCTTGGGGTTACAGA	
Stress Response	*HSP70*	67	Forward	CCACTTCATCGCAGAGTTCAAA	AB196460
			Reverse	GCGAACAGCCCTCTTGTTGT	
	*HSP90*	63	Forward	AGGGTCAAGGAGGTGGTCAA	AB196457
			Reverse	AACGAAGAGGGTGATGGGATATC	
Oxidative Stress	*CAT*	79	Forward	GAGGGCAACTGGGACCTTACT	BE669040
			Reverse	GGACGAAGGACGGGAACAG	

β-actin: beta-actin; 18srRNA: 18S ribosomal ribonucleic acid; EF1-α: elongation factor 1 α; TNFα: tumor necrosis factor α; TGFβ: transforming growth factor β; IL: interleukin; CXCL: C-X-C motif chemokine ligand; HSP: heat shock protein; CAT: catalase.

### 2.7 Statistical analyses

The data were analyzed using RStudio statistical software version 3.6.1. Single Ct outliers were removed when the sample values were larger than [(standard deviation) *2 ± average] in each dietary group. Shapiro-Wilk normality test and Levene's Test for Homogeneity of Variance were performed to assess the normality and variance homogeneity, respectively, in order to meet the assumptions of performing ANOVA tests. For normal data, the R function “aov” was used to perform one-way and two-way ANOVA tests, to determine the significance of lipid and protein as fixed effects on fish growth performance, body indices and relative gene expression. No significant lipid x protein interactions were found, thus it was removed from the model (y = a + b). One-way ANOVA test was also used to investigate the significance between diets. For non-normal data, a Kruskal-Wallis test was performed. Pairwise comparisons were conducted using Tukey Test for normal data, and Wilcoxon rank sum test for non-normal data, with the p-value-adjustment method of “BH” to correct for multiple testing. A P-value < 0.05 was considered as significant.

## 3 Results

### 3.1 Growth performance and relative body indices

**Fig. 1:**
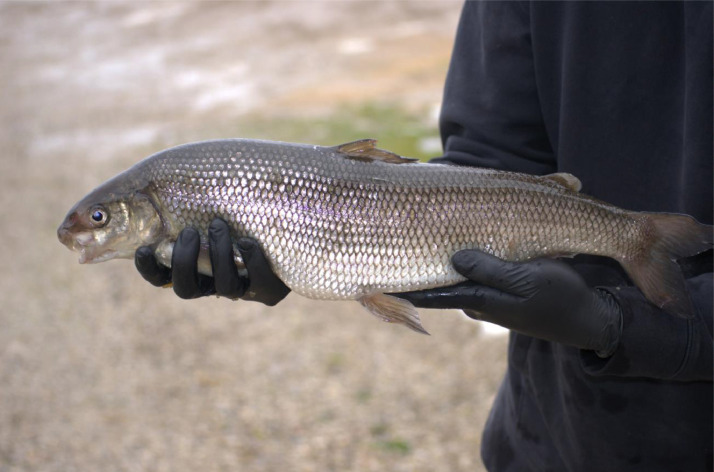
Photo of a juvenile lake whitefish (*Coregonus clupeaformis*).

Lake whitefish fed the HPHL diet had the highest growth and lowest FCR followed by the LPHL diet (Table 3 and Fig. 1). Lipid had a significant effect on WG (*P* < 0.001), SGR (*P =* 0.001), TGC (*P =* 0.002), and FCR (*P =* 0.028). However, no significant effect of lipid levels was found on VSI (P = 0.187) although effect of diet alone was found (*P =* 0.037), where fish fed the HPLL diet had a lower VSI than the BCC diet (*P =* 0.042). Lake whitefish fed diets with higher lipid also resulted in significantly higher FI (*P =* 0.025) and HSI (*P =* 0.025). No significant effects (P>0.05) of protein level (insect inclusion) were found when evaluating the growth performance and feed utilization of lake whitefish.

**Table 3:** Means and P-values for the effects of diet, lipid and protein levels on the growth performance and feed utilization of lake whitefish over 16 weeks.

	BCC	LPHL	LPLL	HPHL	HPLL	SE	1-way ANOVA P-values	2-way ANOVA P-values*	
							Diet	Lipid	Protein
SGR	0.21	0.17	0.12	0.20	0.14	0.04	0.063	**0.001**	0.859
TGC	0.061^a^	0.049^ab^	0.033^b^	0.057^ab^	0.040^ab^	0.012	**0.042**	**0.002**	1.000
WG	78.21^a^	62.92^acd^	41.71^b^	74.07^ad^	51.38^bc^	15.61	**0.001**	**0.0001**	0.716
FI	133.86	124.71	107.82	120.82	96.28	25.36	0.440	**0.025**	0.289
FCR	1.70	1.98	2.56	1.63	1.88	0.40	0.068	**0.028**	0.651
VSI	7.63^a^	6.39^ab^	6.53^ab^	6.76^ab^	6.34^b^	0.63	**0.037**	0.187	0.372
HSI	0.89	0.85	0.69	0.76	0.70	0.14	0.311	**0.025**	0.768
LSI	2.05	1.57	1.60	1.65	1.53	0.34	0.358	0.378	0.439

BCC: Bluewater commercial control; LPHL: low protein + high lipid diet; LPLL: low protein + low lipid diet; HPHL: high protein + high lipid diet; HPLL: high protein + low lipid diet; SE: pooled standard error; SGR: specific growth rate; TGC: thermal growth coefficient; WG: weight gain; FI: feed intake; FCR: feed conversion ratio; VSI: viscerosomatic index; HSI: hepatosomatic index; LSI: liposomatic index.

⁎ Different lowercase letters show significant differences between dietary groups (P < 0.05). Bold number indicate significant effect (P < 0.05), where n=3 per diet (except n=6 for VSI, HSI and LSI).

### 3.2 Plasma biochemistry

**Fig. 2:**
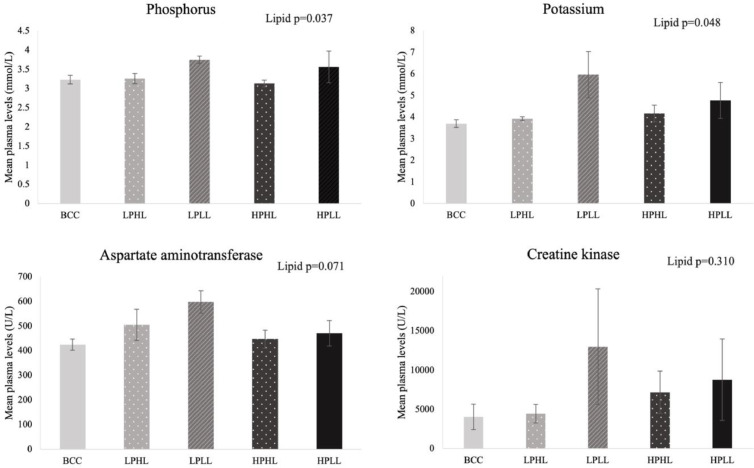
*Plasma biochemistry levels of phosphorus, potassium, aspartate aminotransferase and creatine* kinase (mean ± standard error) of lake whitefish fed Bluewater commercial control (BCC), low protein high lipid (LPHL), low protein low lipid (LPLL), high protein high lipid (HPHL) or high protein low lipid (HPLL). Calibrated using the control LPHL diet, where *n* = 3 per diet.

Lake whitefish fed with lower lipid diets were found to have a significantly higher phosphorus (*P =* 0.037) and potassium (*P =* 0.048) plasma concentrations. Additionally, low lipid diets led to a slight increase in aspartate aminotransferase (*P =* 0.071) and a slight decrease in amylase (*P =* 0.099), although not significant. However, no significant effects of diet and protein/insect inclusion (P>0.05) were found on other plasma biochemistry parameters (Table 4 and Fig. 2).

**Table 4:** Means and P-values for the effects of diet, protein and lipid levels, and insect inclusion on the plasma biochemistry parameters of lake whitefish at 16 weeks.

	BCC	LPHL	LPLL	HPHL	HPLL	SE	1-way ANOVA P-values	2-way ANOVA P-values*	
							Diet	Protein	Lipid
Calcium	2.43	2.36	2.29	2.36	2.32	0.04	0.837	0.968	0.322
Phosphorus	3.23	3.26	3.75	3.13	3.56	0.10	0.263	0.607	**0.037**
Total Protein	40.00	40.00	42.00	38.33	40.67	0.67	0.599	0.610	0.174
Albumin	19.33	19.67	19.00	18.67	19.67	0.32	0.869	0.850	0.677
Globulin	20.67	20.33	23.00	19.67	21.00	0.51	0.329	0.334	0.110
Albumin/Globulin ratio	0.94	0.97	0.83	0.95	0.94	0.02	0.362	0.332	0.270
Glucose	4.57	4.63	4.57	4.67	4.63	0.08	0.995	0.910	0.934
Cholesterol	6.38	7.20	6.74	6.45	7.10	0.20	0.690	0.585	0.290
Aspartate aminotransferase	424.00	505.00	597.00	446.67	470.33	23.72	0.147	0.552	0.071
Creatine kinase	4051	4458	12977	7183	8765	1841	0.673	0.843	0.310
Amylase	30.67	23.00	11.00	23.67	15.00	3.57	0.488	0.930	0.099
Lactate dehydrogenase	1834	2528	3397	2312	2656	260	0.480	0.975	0.134
Bile acid	37.33	31.33	44.33	18.33	48.00	6.40	0.588	0.784	0.209
Glutamate dehydrogenase	40.00	31.67	50.33	42.33	35.67	3.15	0.444	0.334	0.803
Sodium	149	147	143	148	143	2	0.748	0.959	0.184
Potassium	3.70	3.93	5.97	4.17	4.77	0.32	0.164	0.530	**0.048**
Chlorine	120	119	116	121	116	1	0.666	0.941	0.139
Carbon dioxide	10.67	10.00	10.00	10.33	10.00	0.30	0.957	0.929	0.506
Lipase	5.67	6.00	6.33	6.33	6.00	0.15	0.596	0.883	0.852
Uric acid	12.00	12.00	12.00	12.00	14.00	0.40	0.406	0.472	0.472
Urea	0.33	0.30	0.40	0.20	0.30	0.03	0.145	0.111	0.331

BCC: Bluewater commercial control; LPHL: low protein + high lipid diet; LPLL: low protein + low lipid diet; HPHL: high protein + high lipid diet; HPLL: high protein + low lipid diet; SE: pooled standard error.

⁎ Bold number indicate significant P-value < 0.05. No significant results were found for a lipid*protein interaction, where n=3 for each diet.

### 3.3 Gene expression

**Fig. 3:**
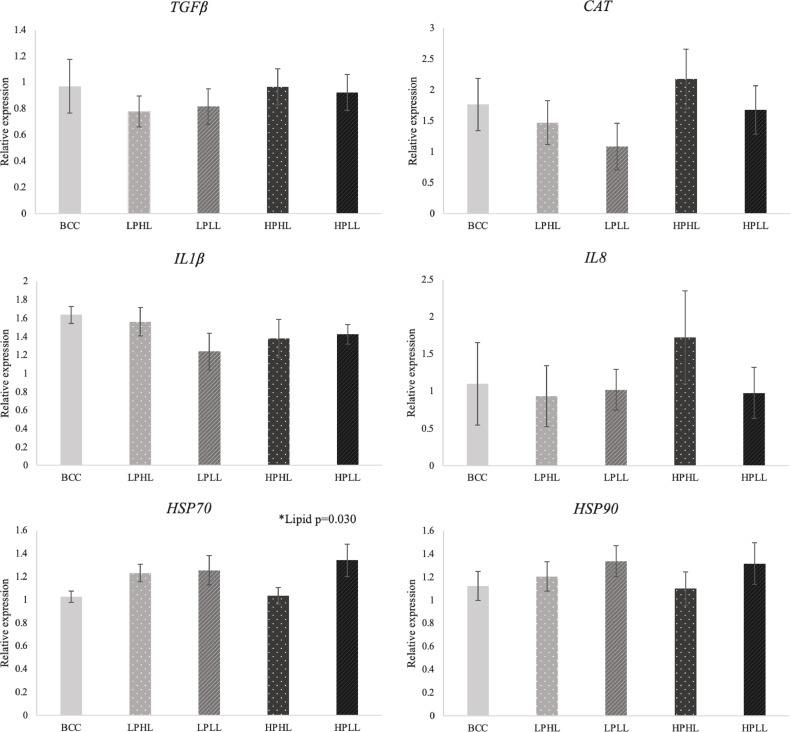
Hepatic gene expression relative to β-actin and EF-1α (mean ± standard error) of lake whitefish fed Bluewater commercial control (BCC), low protein high lipid (LPHL), low protein low lipid (LPLL), high protein high lipid (HPHL) or high protein low lipid (HPLL). *IL8* is also referred to as *CXCL8*. Calibrated using the control LPHL diet, where n=9 per diet (TGFβ: transforming growth factor-β; IL: interleukin; CXCL: C-X-C motif chemokine ligand; HSP: heat shock protein; CAT: catalase).

Dietary lipid significantly (*P =* 0.030) affected the hepatic gene expression of *HSP70*, which were lower (*P =* 0.044) in fish fed high lipid diets (Table 5 and Fig. 3). No significant effects of protein/insect inclusion or lipid*protein interactions were found on the expression of *TGFβ, IL1β, IL8 (CXCL8), HSP70, HSP90* and *CAT* genes (*P* > 0.05).

**Table 5:** P-values for the effects of diet, protein and lipid levels, and insect inclusion on the relative gene expression in the liver of lake whitefish.

	1-way ANOVA P-values	2-way ANOVA P-values	
	Diet	Protein	Lipid
*TGFβ*	0.853	0.509	0.733
*IL1β*	0.601	0.683	0.461
*IL8 (CXCL8)*	0.872	0.707	0.826
*HSP70*	0.117	0.386	**0.030**
*HSP90*	0.606	0.956	0.268
*CAT*	0.436	0.455	0.335

Note: *TGFβ*: transforming growth factor-β; *IL*: interleukin; CXCL: C-X-C motif chemokine ligand; *HSP*: heat shock protein; *CAT*: catalase.

P-values from linear fixed effects models with fixed effects of diet, protein, lipid, and insect. Bold number indicate significant effect (p < 0.05), where n=9 per diet.

## 4 Discussion

The main objective of this study was to investigate the effects of dietary protein:lipid ratios on the growth, feed conversion, body indices, plasma biochemistry, and the hepatic gene expression of lake whitefish after a 16-week feeding trial. This study provided important insights that establish baseline information for future research on lake whitefish since literature on culturing this species is scarce and drastically needed to improve production and expand the diversity of species produced in the aquaculture industry.

### 4.1 Growth performance improved when fed high protein and high lipid diets

Lake whitefish fed the BCC and HPHL diets resulted in significantly increased growth performance compared to fish fed the other diets (Table 3). However, higher VSI was found in fish fed the BCC diet, which is not beneficial for both the farmer and consumer due to reduced fillet yield. Increased deposition of lipid as fat in the viscera is not optimal as this is removed during fish processing. Deposition in the muscle is preferred as this will contribute to an increase in fillet yield. Other health effects, such as fatty liver disease, may also arise due to high VSI [21]. This was reflected in fish fed high lipid diets which had a higher HSI. While this may be a concern, no data demonstrating the normal range of HSI in lake whitefish currently exists. It was surprising to find a significant effect of lipid on VSI without a significant effect on LSI. Our findings indicate that increased lipid in the diet resulted in higher deposition of fat in the organs (intra-organ adipocytes), such as the liver, but not fat around the organs (visceral adipocytes). Similar findings have been found in Atlantic salmon and rainbow trout fed high lipid diets that resulted in higher VSI and HSI parameters, regardless of the lipid source [22], [23], [24].

The SGR, TGC and WG were found to be the lowest in fish fed LPLL diet. Previous research by Mock et al. [25] has found that WG, FCR, SGR and VSI in Atlantic salmon (*Salmo salar*) were similar and no significant differences were found between fish fed HPLL (40% protein and 33% lipid) and LPHL (36% protein and 36% lipid) diets; however, WG was found to be significantly different between HPLL and LPHL diets in the present study. Similar results were found when Senegalese sole (*Solea senegalensis*) were fed a LPLL diet (45% protein and 8% lipid), which resulted in lower WG than fish fed a diet containing 55% protein and 16% lipid [26]. In contrast, lower WG and SGR were observed in Atlantic salmon fed high lipid diets compared to low lipid diets, although the fat content was on the higher end of the spectrum (21 vs 34% total lipid) [23]. The significant decrease in growth performance in the LPLL group observed in the present study suggests that inclusion of 48% of protein and 12% lipid in the diet was not sufficient to support the optimal growth performance of lake whitefish.

Protein requirements in fish are also important for growth, considering most carnivorous fish (e.g. salmonids) require a diet including 30-55% of crude protein to provide sufficient levels of amino acids for optimal growth, development and health [27]. Previous studies have found that Atlantic salmon can still achieve optimal growth when fed diets with low protein if they have high inclusion of dietary lipids [28]. This is based on a concept called “protein-sparing effect” where lipid is more energy dense, thus the preferred energy source in diets for fish since protein metabolism and transport is energetically demanding [22,29,30]. In the present study, SGR, TGC and WG were the third highest in the LPHL control dietary group after the BCC and HPHL diets, which is in line with these previous studies on Atlantic salmon.

Dietary lipids are an important source of energy for salmonid fishes. Research has demonstrated that feed efficiency and growth performance can be improved when up to 30% of the diet is comprised of lipid [31]. In the present study, WG, FCR, FI and SGR were found to be significantly improved in lake whitefish fed with high lipid diets. Similar results were found in Atlantic salmon fed between 6.6% and 29.4% of dietary lipid, where WG and SGR increased with lipid levels up to 29.4% [32]. Similarly, Atlantic salmon fed diets with a low or high lipid level (18 or 23%) and a low or high n-3 LC-PUFA level (0.7 or 1.4 %) resulted in improved final weight for fish fed high lipid and high n-3 LC-PUFA diets [22]. Other studies have also found similar effects on rainbow trout and shrimp (*Penaeus monodon*) [33,34]. Higher growth performance of lake whitefish reflects previous studies on salmonids and other aquatic species.

### 4.2 Plasma biochemistry diminished when fed low lipid diets

Plasma phosphorus concentrations were found to be higher in lake whitefish fed with low lipid diets, which may be due to lower weight gain in these fish and consequently more concentrated levels in the blood (Table 4 and Fig. 2). Huyben et al. [35] found that low lipid diets resulted in lower WG and increased digestibility of phosphorus for Atlantic salmon. The high concentration of phosphorus in the plasma may be an end-product of metabolizing protein instead of lipid, as noted above, which is less energy dense. Despite higher levels of protein in the diet and insect inclusion, total protein levels and urea in the plasma were not significantly affected. In contrast, a previous study also found that blood urea concentrations were elevated in barramundi (*Lates calcarifer*) fed 30% BSFL oil inclusion [36], whereas the present study only fed 5% full-fat BSFL. Also, rainbow trout fed with 6.6% of BSFL resulted in significantly lower plasma phosphorus and higher magnesium concentration compared to fish fed the control diet [37]. A meta-analysis by Tran et al. [38] showed that increasing levels of insects in the diet reduced solid phosphorus waste, which is especially beneficial in freshwater environments to reduce the potential for harmful algal blooms. Potentially lower content or higher digestibility of phosphorus in insects have been suggested [39], which may have caused lower plasma levels in the present study.

In the present study, low lipid diets resulted in negative effects on several plasma biochemical profiles that include aspartate aminotransferase and potassium. Low lipid diets had an increased tendency of plasma aspartate aminotransferase, which is an indicator of disease and liver dysfunction [40]. Lastly, plasma potassium concentrations were also higher in fish fed the low lipid diet, which has been shown to be a secondary indicator of damage due to stress [41]. Lower growth and adverse effects on plasma biochemistry confirm that low lipid diets at 12% inclusion are too low and are not recommended for lake whitefish.

Furthermore, plasma creatine kinase (CK) concentration was not significantly affected by protein, lipid, BSFL additives, or different dietary groups in this study (Table 4 and Fig. 2). Similarly, Weththasinghe et al. [39] found that CK levels were not affected in Atlantic salmon when fed diets 20% full-fat BSFL and 15% defatted BSFL and did not lead to damage in muscle tissues since CK is found to be concentrated in muscle and heart tissues. Additionally, plasma glucose concentrations were unchanged in this study among different dietary groups, indicating an absence of a dietary stressor and that glucose was not a significant source of energy for lake whitefish during this trial [42]. However, the LPLL diet in this study has a relatively higher plasma CK concentration compared to other dietary groups (even though not significant), which may be an indication of damage in muscle and heart tissues for lake whitefish.

### 4.3 Gene expression downregulated by high lipid diets

Heat shock proteins (HSPs), such as HSP70 and HSP90, function as helper molecules to regulate the metabolic activities, immune signalling and inflammatory processes of the cell and are produced when the cells are exposed to stress [43]. In this study, the relative expression of *HSP70* was upregulated in the liver of lake whitefish fed with the low lipid (12%) diet and downregulated when fed the high lipid (18%) diet (Table 5 and Fig. 3). Similarly, Jirsa et al. [44] found a positive correlation between the dietary lipid levels and *HSP70* relative expression in the white muscle of juvenile white seabass (*Atractoscion nobilis*). Hepatic *HSP70* expression in white seabass was upregulated with increasing dietary lipid levels from 10% to 14%, however, *HSP70* expression was then downregulated in fish fed with 16% and 18% dietary lipid, which suggested that dietary lipid level more than 14% tends to downregulate *HSP70* expression [44]. Lake whitefish fed the HPHL diet in the current study grew faster than other groups and FCR was significantly improved, which suggests that the fish directed their energy towards growth rather than maintaining the *HSP70/90* pathway. We were surprised to find the relative expression of *HSP90* in the liver of lake whitefish was not significantly affected by either dietary protein or lipid levels in this study. However, similar trends of downregulated *HSP90* expression in fish fed high lipid diets (18%) was observed, but was not significant. More research is required to investigate if the downregulation of *HSP70/90* expression in lake whitefish is beneficial to their health and growth performance or a remnant of higher weight gain.

Similar to previous studies on Atlantic salmon, barramundi, largemouth bass, yellow catfish and zebrafish [36,39,[45], [46], [47], [48]], there was no significant effect on the expression of *HSP70/90* in lake whitefish fed high protein/insect diets. While a few studies have demonstrated an effect of insect inclusion on *HSP* expression in Atlantic salmon, European seabass (*Dicentrarchus labrax*) and common carp (*Cyprinus carpio*) [17,49,50], these studies fed higher inclusion levels of insect meal, which may explain the lack of significant effects on *HSP* expression in the present study. The inclusion of an earlier time point in future studies would also be beneficial since the pro-inflammatory gene expression would be induced immediately after a stimulus and recovers relatively fast as found in Atlantic salmon [4], which leads to the possibility that this early inflammatory regulation had already diminished at the end of the 112 day trial.

Upregulation of pro-inflammatory cytokines is considered beneficial to aid the host in defending against pathogens and stressors [51]. In addition, previous studies have suggested that feeding insects, specifically chitin in the exoskeleton, to fish may benefit the host by upregulating pro-inflammatory cytokines and improving immune function and disease resistance [12,49]. The results from the present study showed that the relative expression of *IL-1β, IL-8 (CXCL8)* and *TGF-β* were not significantly affected by 5% of insect meal (Table 5 and Fig. 3). Similarly, Kumar et al. [16] found that *IL-8* expression in the intestine of rainbow trout was not affected by 8% and 16% replacement of fish meal with BSFL meal. However, contradictory results were found by Abdel-Latif et al. [17], who observed upregulation of *IL-1β* in the liver of European seabass when fed with 25%, 35% and 50% of BSFL meal, which was positively correlated with the level of insect meal. Stenberg et al. [45] found upregulation of *IL-1β* and *IL-8* in the head kidney of Atlantic salmon fed 66% BSFL. Hender et al. [36] also found upregulation of *IL-1β* in the liver of barramundi when fed 22% of BSF protein and 1% BSF oil. These results suggest that a higher percentage (e.g. >20%) of BSFL meal replacement of fish meal is required to significantly upregulate expression of cytokine genes of fish, thus, the 5% inclusion of insect meal in our study was not sufficient to induce a significant change to the innate immune response. However, another theory is that the inclusion is not high enough to alter the gut microbiome, which may indirectly affect the immune response as found in a previous rainbow trout study [12].

This study found that the relative expressions of *IL-1β, IL-8* and *TGF-β* were not significantly different among the five dietary groups with varied protein:lipid ratios. However, Jin et al. [6] found that the lysozyme activity and reaction oxygen species (ROS) production in the non-specific immune system of juvenile grass carp (*Ctenopharyngodon idella)* were positively correlated with dietary lipid levels. The lack of lipid effects on gene expression of these cytokines may be due to the small variations of dietary lipid (18 and 12%) in this study, which is not distinct enough to induce a change in cytokine expression in lake whitefish. In addition, lack of significant effects on gene expression may be because lake whitefish are pseudotetraploid since salmonid fishes are believed to have undergone a rapid radiation between 25 and 100 million years ago following a tetraploidization event (doubling of chromosomes) that characterizes the Salmonidae family [52]. Therefore, it is possible that the paralogs we selected in our study were not influenced by different dietary treatments whereas other paralogs may have been.

Catalase (CAT) is an important enzyme that plays key roles in antioxidant defense mechanism to break down toxic reactive oxygen species that are unbalanced and produced under stressful environmental conditions [53]. In our study, the relative expression of *CAT* was not significantly affected by either protein:lipid ratio or insect meal addition. Interestingly, a positive correlation was recorded in largemouth bass (*Micropterus salmoides*) between the relative expression of *CAT* and the increasing lipid levels from 3.3 to 18.1%, and a significantly downregulation was also recorded when the dietary lipid level increased to 23.3% [54]. Similar results were found in the study by Zhou et al. [55], where *CAT* expression was upregulated when dietary lipid levels increased from 5% to 10%. However, as the dietary lipid level increased up to 20%, *CAT* expression was downregulated, indicating a possible hepatic dysfunction when fish consume a high lipid diet. As mentioned above, the difference in dietary lipid may have been too small to produce a significant effect on *CAT* expression.

## 5 Conclusions

In conclusion, our hypothesis that the high lipid, high protein diets and the addition of BSFL as a feed additive improves the growth performance, plasma biochemistry and the innate immune response of lake whitefish was rejected. Our results showed the growth performance of lake whitefish was significantly increased when fed with BCC diet and HPHL diets; however, VSI was lower in fish fed HLHP suggesting this is more optimal for non-viscera (fillet) weight gain. We found that feeding fish with higher dietary lipid level (18%) resulted in significantly lower *HSP70* relative expression, which may indicate less cellular damage and repair. No effect of dietary lipid and protein were found on hepatic expression of genes related to innate immunity and oxidative stress. Fish fed the low lipid diets showed increased levels of plasma phosphorus, aspartate aminotransferase and potassium, which indicates poor growth and physiological stress. In addition, 5% BSFL as a feed additive did not influence any growth performance, plasma biochemistry or gene expression parameters, which suggests 5% inclusion of BSFL was too low and higher levels are recommended in future studies. Overall, we recommend HPHL diet for lake whitefish based on improved growth performance, low viscera weight, improved plasma biochemistry and downregulation of cellular repair genes.

## Author contributions

DH was involved in conceptualization, supervision, project administration and funding acquisition. DH, MC, RL and YC were involved in formal analysis and investigation. YC, US and NK were involved in formal analysis. YC was involved in writing the original manuscript and all authors were involved in review and editing.

## Funding

This research was funded by OMAFRA (Ontario Ministry of Agriculture, Food and Rural Affairs) Alliance Tier II program (UG-T2-2020-101365) and the University of Guelph.

## Declaration of Competing Interest

The authors declare that they have no known competing financial interests or personal relationships that could have appeared to influence the work reported in this paper.

## Data Availability

Data will be made available on request.

## References

[1] Government of Ontario, “Lake whitefish (Opeongo lake large and small-bodied populations).” http://www.ontario.ca/page/lake-whitefish-opeongo-lake-large-and-small-bodied-populations (accessed Apr. 12, 2023).

[2] Ebener M.P., Kinnunen R.E., Schneeberger P.J., Mohr L.C., Hoyle J.A., Peeters P. (2008). Management of commercial fisheries for lake whitefish in the Laurentian Great Lakes of North America. International Governance of Fisheries Ecosystems.

[3] Henry M., Fountoulaki E. (2014). Optimal dietary protein/lipid ratio for improved immune status of a newly cultivated Mediterranean fish species, the shi drum *Umbrina cirrosa, L*. Fish Shellfish Immunol..

[4] Huyben D. (2023). Steroidogenic and innate immune responses in Atlantic salmon are influenced by dietary total lipid, long chain polyunsaturated fatty acids and dissolved oxygen. Aquaculture.

[5] Gutiérrez S., Svahn S.L., Johansson M.E. (2019). Effects of Omega-3 fatty acids on immune cells. Int. J. Mol. Sci..

[6] Jin Y. (2013). Dietary lipid requirement on non-specific immune responses in juvenile grass carp (*Ctenopharyngodon idella*). Fish Shellfish Immunol..

[7] Kiron V. (2012). Fish immune system and its nutritional modulation for preventive health care. Anim. Feed Sci. Technol..

[8] Henry M., Gasco L., Piccolo G., Fountoulaki E. (2015). Review on the use of insects in the diet of farmed fish: Past and future. Anim. Feed Sci. Technol..

[9] Nogales-Mérida S. (2019). Insect meals in fish nutrition. Rev. Aquac..

[10] Randazzo B. (2021). Hermetia illucens and poultry by-product meals as alternatives to plant protein sources in gilthead seabream (*Sparus aurata*) diet: a multidisciplinary study on fish gut status. Animals.

[11] Aragão C., Gonçalves A.T., Costas B., Azeredo R., Xavier M.J., Engrola S. (2022). Alternative proteins for fish diets: implications beyond growth. Animals.

[12] Huyben D., Vidaković A., Hallgren S.Werner, Langeland M. (2019). High-throughput sequencing of gut microbiota in rainbow trout (*Oncorhynchus mykiss*) fed larval and pre-pupae stages of black soldier fly (*Hermetia illucens*). Aquaculture.

[13] Józefiak A., Nogales-Mérida S., Mikołajczak Z., Rawski M., Kierończyk B., Mazurkiewicz J. (2019). The utilization of full-fat insect meal in rainbow trout (*Oncorhynchus mykiss*) nutrition: the effects on growth performance, intestinal microbiota and gastrointestinal tract histomorphology. Ann. Anim. Sci..

[14] Rimoldi S., Gini E., Iannini F., Gasco L., Terova G. (2019). The effects of dietary insect meal from hermetia illucens prepupae on autochthonous gut microbiota of rainbow trout (*Oncorhynchus mykiss*. Animals.

[15] Bruni L., Milanović V., Tulli F., Aquilanti L., Parisi G. (2022). Effect of diets containing full-fat *Hermetia illucens* on rainbow trout microbiota: A dual cultivation-independent approach with DGGE and NGS. Aquaculture.

[16] Kumar V. (2021). Insect (black soldier fly, *Hermetia illucens*) meal supplementation prevents the soybean meal-induced intestinal enteritis in rainbow trout and health benefits of using insect oil. Fish Shellfish Immunol..

[17] Abdel-Latif H.M.R. (2021). Black soldier fly (*Hermetia illucens*) larvae meal in diets of European seabass: effects on antioxidative capacity, non-specific immunity, transcriptomic responses, and resistance to the challenge with *Vibrio alginolyticus*. Fish Shellfish Immunol..

[18] A. Foey and S. Picchietti, “Immune defences of teleost fish,” 2014, pp. 14–52. doi: 10.1002/9781118897263.ch2.

[19] Xie F., Xiao P., Chen D., Xu L., Zhang B. (2012). miRDeepFinder: a miRNA analysis tool for deep sequencing of plant small RNAs. Plant Mol. Biol..

[20] Vandesompele J. (2002). Accurate normalization of real-time quantitative RT-PCR data by geometric averaging of multiple internal control genes. Genome Biol..

[21] Xue M. (2022). Mechanism analysis of metabolic fatty liver on largemouth bass (*Micropterus salmoides*) based on integrated lipidomics and proteomics. Metabolites.

[22] Huyben D., Grobler T., Matthew C., Bou M., Ruyter B., Glencross B. (2021). Requirement for omega-3 long-chain polyunsaturated fatty acids by Atlantic salmon is relative to the dietary lipid level. Aquaculture.

[23] Bendiksen E.Å., Berg O.K., Jobling M., Arnesen A.M., Måsøval K. (2003). Digestibility, growth and nutrient utilisation of Atlantic salmon parr (*Salmo salar L*.) in relation to temperature, feed fat content and oil source. Aquaculture.

[24] Moccia R.D., Gurure R.M., Atkinson J.L., Vandenberg G.W. (1998). Effects of the repartitioning agent ractopamine on the growth and body composition of rainbow trout, *Oncorhynchus mykiss* (Walbaum), fed three levels of dietary protein. Aquac. Res..

[25] Mock T.S. (2019). The impact of dietary protein: lipid ratio on growth performance, fatty acid metabolism, product quality and waste output in Atlantic salmon (*Salmo salar*). Aquaculture.

[26] Guerreiro I., Peres H., Castro-Cunha M., Oliva-℡es A. (2012). Effect of temperature and dietary protein/lipid ratio on growth performance and nutrient utilization of juvenile Senegalese sole (*Solea senegalensis*). Aquac. Nutr..

[27] Bowyer J.N., Qin J.G., Stone D.A.J. (2013). Protein, lipid and energy requirements of cultured marine fish in cold, temperate and warm water. Rev. Aquac..

[28] Glencross B.D. (2009). Exploring the nutritional demand for essential fatty acids by aquaculture species. Rev. Aquac..

[29] Karalazos V., Bendiksen E.Å., Bell J.G. (2011). Interactive effects of dietary protein/lipid level and oil source on growth, feed utilisation and nutrient and fatty acid digestibility of Atlantic salmon. Aquaculture.

[30] Solberg C. (2004). Influence of dietary oil content on the growth and chemical composition of Atlantic salmon (*Salmo salar*). Aquac. Nutr..

[31] Ghanawi J., Roy L., Davis D.A., Saoud I.P. (2011). Effects of dietary lipid levels on growth performance of marbled spinefoot rabbitfish *Siganus rivulatus*. Aquaculture.

[32] Meng Y. (2019). Effects of dietary lipid levels on sub-adult triploid rainbow trout (*Oncorhynchus mykiss*): 1. Growth performance, digestive ability, health status and expression of growth-related genes. Aquaculture.

[33] Watanabe T. (1982). Lipid nutrition in fish. Comp. Biochem. Physiol. Part B Comp. Biochem..

[34] Glencross B., Smith D.M., Thomas M.R., Williams K.C. (2002). Optimising the essential fatty acids in the diet for weight gain of the prawn, *Penaeus monodon*. Aquaculture.

[35] Huyben D., Matthew C., Muñoz-Lopez P., Ruyter B., Glencross B. (2021). Hypoxia does not change responses to dietary omega-3 long-chain polyunsaturated fatty acids, but rather reduces dietary energy demand by Atlantic salmon. Aquac. Nutr..

[36] Hender A., Siddik M.A.B., Howieson J., Fotedar R. (2021). Black soldier fly, *Hermetia illucens* as an alternative to fishmeal protein and fish oil: impact on growth, immune response, mucosal barrier status, and flesh quality of juvenile barramundi, *Lates calcarifer* (Bloch, 1790). Biology.

[37] Dumas A., Raggi T., Barkhouse J., Lewis E., Weltzien E. (2018). The oil fraction and partially defatted meal of black soldier fly larvae (*Hermetia illucens*) affect differently growth performance, feed efficiency, nutrient deposition, blood glucose and lipid digestibility of rainbow trout (*Oncorhynchus mykiss*). Aquaculture.

[38] Tran H.Q., Nguyen T.T., Prokešová M., Gebauer T., Doan H.V., Stejskal V. (2022). Systematic review and meta-analysis of production performance of aquaculture species fed dietary insect meals. Rev. Aquac..

[39] Weththasinghe P., Lagos L., Cortés M., Hansen J.Ø., Øverland M. (2021). Dietary inclusion of black soldier fly (*Hermetia Illucens*) Larvae meal and paste improved gut health but had minor effects on skin mucus proteome and immune response in Atlantic salmon (*Salmo Salar*). Front. Immunol..

[40] Ndrepepa G. (2021). Aspartate aminotransferase and cardiovascular disease—a narrative review. J. Lab. Precis. Med..

[41] Öner M., Atli G., Canli M. (2008). Changes in serum biochemical parameters of freshwater fish *Oreochromis niloticus* following prolonged metal (Ag, Cd, Cr, Cu, Zn) exposures. Environ. Toxicol. Chem..

[42] D. Suljević, A. Alijagić, E. Islamagić, A. Alijagić, and E. Islamagić, “Temporal influence of spawning on serum biochemical parameters in brown trout *salmo trutta* (Teleostei: salmonidae)” 2023.

[43] Roberts R.J., Agius C., Saliba C., Bossier P., Sung Y.Y. (2010). Heat shock proteins (chaperones) in fish and shellfish and their potential role in relation to fish health: a review. J. Fish Dis..

[44] Jirsa D., Deng D.F., a. Davis D., Wang W.F., s. o. Hung S., Drawbridge M. (2013). The effects of dietary lipid levels on performance and heat-shock protein response of juvenile white seabass, *Atractoscion nobilis*. Aquac. Nutr..

[45] Stenberg O.K. (2019). Effect of dietary replacement of fish meal with insect meal on *in vitro* bacterial and viral induced gene response in Atlantic salmon (*Salmo salar*) head kidney leukocytes. Fish Shellfish Immunol..

[46] Gu J. (2022). A study of the potential effect of yellow mealworm (*Tenebrio molitor*) substitution for fish meal on growth, immune and antioxidant capacity in juvenile largemouth bass (*Micropterus salmoides*). Fish Shellfish Immunol..

[47] Su J. (2017). Effects of dietary Tenebrio molitor meal on the growth performance, immune response and disease resistance of yellow catfish (*Pelteobagrus fulvidraco*. Fish Shellfish Immunol..

[48] Zarantoniello M. (2019). A six-months study on Black Soldier Fly (*Hermetia illucens*) based diets in zebrafish. Sci. Rep..

[49] Li Y., Kortner T.M., Chikwati E.M., Munang'andu H.M., Lock E.J., Krogdahl Å. (2019). Gut health and vaccination response in pre-smolt Atlantic salmon (*Salmo salar*) fed black soldier fly (*Hermetia illucens*) larvae meal. Fish Shellfish Immunol..

[50] Ji H., Zhang J.L., Huang J.Q., Cheng X.F., Liu C. (2015). Effect of replacement of dietary fish meal with silkworm pupae meal on growth performance, body composition, intestinal protease activity and health status in juvenile Jian carp (*Cyprinus carpio var. Jian*). Aquac. Res..

[51] Xiang J. (2020). Growth performance, immunity and intestinal microbiota of swamp eel (*Monopterus albus*) fed a diet supplemented with house fly larvae (*Musca domestica*. Aquac. Nutr..

[52] Johnson K.R., Wright J.E., May B. (1987). Linkage relationships reflecting ancestral tetraploidy in salmonid fish. Genetics.

[53] Lushchak V.I. (2011). Environmentally induced oxidative stress in aquatic animals. Aquat. Toxicol..

[54] Guo J., Zhou Y., Zhao H., Chen W.Y., Chen Y.J., Lin S.M. (2019). Effect of dietary lipid level on growth, lipid metabolism and oxidative status of largemouth bass, *Micropterus salmoides*. Aquaculture.

[55] Zhou Y.L., Guo J.L., Tang R.J., Ma H.J., Chen Y.J., Lin S.M. (2020). High dietary lipid level alters the growth, hepatic metabolism enzyme, and anti-oxidative capacity in juvenile largemouth bass *Micropterus salmoides*. Fish Physiol. Biochem..

